# Poly[[tris­(μ_2_-4,4′-bipyridine *N*,*N*′-di­oxide)hexa­nitratodieuropium(III)] dichloro­methane disolvate]

**DOI:** 10.1107/S1600536810033246

**Published:** 2010-08-21

**Authors:** Adam J. Dillner, Cassandra P. Lilly, Jacqueline M. Knaust

**Affiliations:** a Allegheny College, 520 North Main St., Meadville, PA 16335, USA

## Abstract

The title one-dimensional coordination network, {[Eu_2_(NO_3_)_6_(C_10_H_8_N_2_O_2_)_3_]·2CH_2_Cl_2_}_*n*_, is isostructural with the previously reported Tb and Tl coordination networks and to its Gd analog. The Eu^III^ cation is coordinated in a distorted tricapped trigonal-prismatic fashion by nine O atoms from three bridging 4,4′-bipyridine *N*,*N*′-dioxide ligands and three chelating nitrate anions. None of the atoms lie on a special position, but there is an inversion center located between the rings of one of the ligands. The network topology is ladder-like, and each ladder inter­acts with six neighboring ladders through C—H⋯O hydrogen bonds. The packing motif of the ladders allows for the formation of channels that run parallel to the *a* axis; these channels are filled with CH_2_Cl_2_ solvent mol­ecules that inter­act with the ladders through C—H⋯O hydrogen bonds.

## Related literature

For the isostructural Tb and Tl coordination networks, see: Long *et al.* (2002 ▶); Moitsheki *et al.* (2006 ▶). For the isostructural Gd coordination network, see: Dillner *et al.* (2010 ▶). For additional discussions on *Ln* ^+3^ (*Ln* = lanthanide) coordination networks with aromatic *N,N*’-dioxide ligands, see: Cardoso *et al.* (2001 ▶); Hill *et al.* (2005 ▶); Long *et al.* (2001 ▶); Sun *et al.* (2004 ▶). For background information on the applications of coordination networks, see: Roswell & Yaghi (2004 ▶); Rosi *et al.* (2003 ▶); Seo *et al.* (2000 ▶).
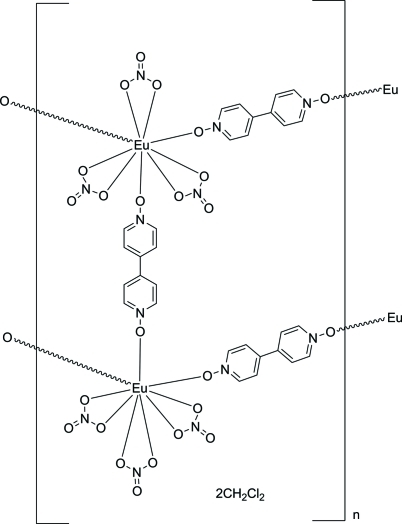


## Experimental

### 

#### Crystal data

[Eu_2_(NO_3_)_6_(C_10_H_8_N_2_O_2_)_3_]·2CH_2_Cl_2_
*M* *_r_* = 1410.38
Triclinic, 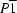

*a* = 7.9841 (5) Å
*b* = 11.5723 (7) Å
*c* = 13.0522 (8) Å
α = 86.013 (1)°
β = 80.255 (1)°
γ = 78.392 (1)°
*V* = 1163.45 (12) Å^3^
*Z* = 1
Mo *K*α radiation
μ = 3.00 mm^−1^
*T* = 100 K
0.44 × 0.38 × 0.32 mm

#### Data collection

Bruker SMART APEX CCD diffractometer
Absorption correction: multi-scan (*SADABS*; Bruker, 2009 ▶) *T*_min_ = 0.278, *T*_max_ = 0.383
13873 measured reflections
7017 independent reflections
6748 reflections with *I* > 2σ(*I*)
*R*_int_ = 0.015

#### Refinement

*R*[*F* ^2^ > 2σ(*F* ^2^)] = 0.020
*wR*(*F* ^2^) = 0.050
*S* = 1.06
7017 reflections
334 parameters
H-atom parameters constrained
Δρ_max_ = 1.30 e Å^−3^
Δρ_min_ = −0.90 e Å^−3^

### 

Data collection: *APEX2* (Bruker, 2009 ▶); cell refinement: *SAINT* (Bruker, 2009 ▶); data reduction: *SAINT*; program(s) used to solve structure: *SHELXS97* (Sheldrick, 2008 ▶); program(s) used to refine structure: *SHELXL97* (Sheldrick, 2008 ▶); molecular graphics: *X-SEED* (Barbour, 2001 ▶); software used to prepare material for publication: *X-SEED*.

## Supplementary Material

Crystal structure: contains datablocks I, global. DOI: 10.1107/S1600536810033246/zl2302sup1.cif

Structure factors: contains datablocks I. DOI: 10.1107/S1600536810033246/zl2302Isup2.hkl

Additional supplementary materials:  crystallographic information; 3D view; checkCIF report

## Figures and Tables

**Table 1:** Hydrogen-bond geometry (Å, °)

*D*—H⋯*A*	*D*—H	H⋯*A*	*D*⋯*A*	*D*—H⋯*A*
C5—H5⋯O7^i^	0.95	2.41	3.081 (2)	128
C9—H9⋯O9^ii^	0.95	2.57	3.286 (2)	132
C12—H12⋯O2^iii^	0.95	2.44	3.309 (2)	152
C16—H16*B*⋯O12^ii^	0.99	2.42	3.242 (3)	140
C16—H16*A*⋯O8	0.99	2.55	3.307 (3)	133
C16—H16*A*⋯O9	0.99	2.50	3.086 (3)	118

Symmetry codes: (i) 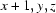; (ii) 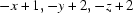; (iii) 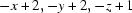.

**Fig. 1.**
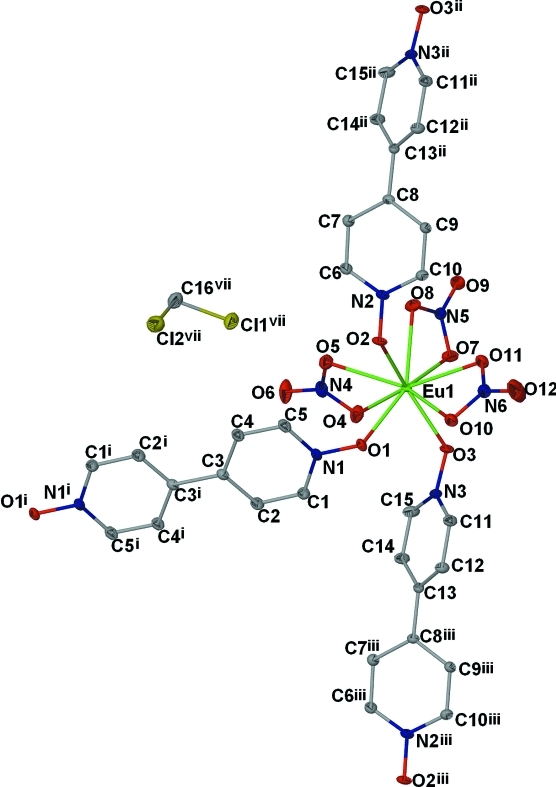
The coordination environment of the Eu+3 cation in the title compound with atom labels and 50% probability displacement ellipsoids. Hydrogen atoms have been omitted for clarity. Color scheme: Nd: green, C: grey, N: blue, O: red, Cl: yellow. Symmetry codes: (i) -x+3, -y+1, -z+1; (ii) x, y, z+1; (iii) x, y, z-1; (vii) -x+2, -y+1, z+2.

**Fig. 2.**
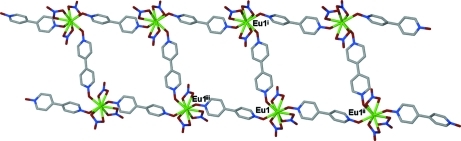
Ladder-like network topology seen in the title compound viewed perpendicular to the (1 2 0) plane. The sides and rungs of the ladder meet at angles of 70.09(<1)° ( Eui—Eu—Euiii) and 108.91(<1)° (Eui—Eu—Euii). Hydrogen atoms and solvate molecules have been omitted for clarity. Color scheme: Nd: green, C: grey, N: blue, O: red. Symmetry codes: (i) -x+3, -y+1, -z+1; (ii) x, y, z+1; (iii) x, y, z-1.

**Fig. 3.**
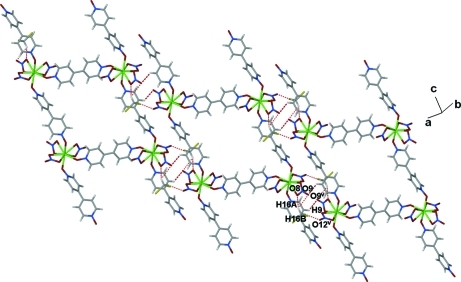
C—H···O hydrogen bonding interactions between 4,4'-bipyridine-N,N'-dioxide ligands and between CH2Cl2 solvate molecules and nitrate anions. These interactions are responsible for linking together ladders that lie in the same plane. Hydrogen bonds are shown as dashed red lines. Color scheme: Nd: green, C: grey, H: white, N: blue, O: red, Cl: yellow. Symmetry code: (v) -x+1, -y+2, -z+2.

**Fig. 4.**
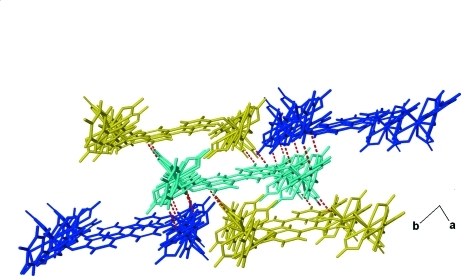
C—H···O hydrogen bonding interactions between 4,4'-bipyridine-N,N'-dioxide ligands, C12—H12···O2vi, and between a 4,4'-bipyridine-N,N'-dioxide ligand and a nitrate anion, C5—H5···O7iv. These interactions link the ladder shown in aqua to the four ladders above and below it that are shown in blue and yellow. Hydrogen bonds are shown as dashed red lines. Symmetry codes: (iv) x+1, y, z; (vi) -x+2, -y+2, -z+1.

**Fig. 5.**
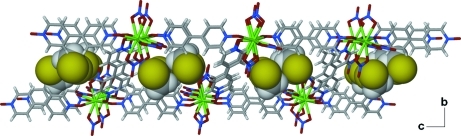
Packing of the title compound viewed along the a-axis with CH2Cl2 solvate molecules represented by van der Waals radii. Color scheme: Nd: green, C: grey, H: white, N: blue, O: red, Cl: yellow.

## supplementary crystallographic information

### Comment

The synthesis of lanthanide coordination networks has been of recent interest due to the potential of the flexible coordination sphere of the Ln^+3^ metal ions to produce coordination networks with new, unusual, or high connectivity topologies (Hill *et al.*, 2005; Long *et al.*, 2001; and Sun *et al.*, 2004). Coordination networks with both a high connectivity topology and an open framework have potential for applications in areas such as absorption, ion exchange, or catalysis (Roswell *et al.*, 2004; Rosi *et al.*, 2003; and Seo *et al.* 2000). Aromatic *N,N*'-dioxide ligands have been attractive candidates for use with Ln^+3^ cations as the O-donor atoms of the ligand are complementary to the hard acid character of the lanthanide cations (Cardoso *et al.,* 2001; Hill *et al.*, 2005; Long *et al.*, 2001; Long *et al.*, 2002; and Sun *et al.*, 2004).

The description of the structure of the title compound is part of a set of consecutive papers on one-dimensional ladder-like coordination networks of the type [Ln_2_(NO_3_)_6_(C_10_H_8_N_2_O_2_)_3_]_n_, with Ln = Eu (this publication) and Gd (Dillner *et al.*, 2010), respectively. Both compounds are also isostructural to the previously reported Tb and Tl coordination networks (Long *et al.*, 2002 and Moitsheki *et al.*, 2006).

The asymmetric unit of the title compound contains one Eu^+3^ cation, one and a half coordinated *4,4*'-bipyridine-*N,N*'-dioxide ligands, three coordinated nitrate anions, and one solvate CH_2_Cl_2_ molecule. None of the atoms lie on a special position, but there is an inversion center located between the rings of one of the ligands (O1, N1, C1-C5). The Eu^+3 ^cation is coordinated in a distorted tricapped trigonal prismatic fashion by nine O atoms (Figure 1). Three bridging *4,4*'-bipyridine-*N,N*'-dioxide ligands contribute three O donor atoms, and three nitrate anions contribute six O donor atoms. The network topology is ladder-like; however the sides and rungs of the ladder meet at angles of 70.09(<1)° ( Eu^i^—Eu—Eu^iii^) and 108.91(<1)° (Eu^i^—Eu—Eu^ii^) forming a parallelogram rather than a square [Symmetry codes: (i) -x+3, -y+1, -z+1; (ii) x, y, z+1; (iii) x, y, z-1] (Figure 2). The ladders run parallel to the *c*-axis and lie in planes that are approximately parallel with the (1 2 0) plane.

Through C-H···O hydrogen bonding interactions the ladders are linked into a three-dimensional structure. Each ladder is linked to two similar ladders that lie in the same plane through four unique C-H···O hydrogen bonds per Eu^+3^ cation (Figure 3). There is one direct interaction between the ladders via a C-H···O hydrogen bond from a *4,4*'-bipyridine-*N,N*'-dioxide ligand of one ladder to the nitrate anion of another ladder, C9—H9···O9^v^ [Symmetry code:(v) -x+1, -y+2, -z+2]. There is also an indirect interaction between the ladders through hydrogen bonding with the CH_2_Cl_2_ solvate molecules. Two O atoms of a nitrate ion hydrogen bond with one of the CH_2_Cl_2_ H atoms, C16—H16A···O8 and C16—H16A···O9; the other H atom of the CH_2_Cl_2_ molecule then hydrogen bonds with an O atoms of a nitrate ion of the neighboring ladder, C16—H16B···O12^v ^[Symmetry code:(v) -x+1, -y+2, -z+2]. The ladders are further linked to two neighboring ladders in the layer above and two in the layer below through hydrogen bonding interactions between *4,4*'-bipyridine-*N,N*'-dioxide ligands, C12—H12···O2^vi^, and between a *4,4*'-bipyridine-*N,N*'-dioxide ligand and a nitrate anion, C5—H5···O7^iv ^[Symmetry codes:(iv) x+1, y, z; (vi) -x+2, -y+2, -z+1] (Figure 4).

Though the Eu^+3^ cation is nine coordinate, the use of the coordinating nitrate counter ion limits the number of bridging *4,4*'-bipyridine-*N,N*'-dioxide ligands resulting in a one-dimensional coordination network rather than a network with a high connectivity topology. However, the packing motif of the ladders allows for the formation of channels that run parallel to the *a*-axis; these channels are filled with the CH_2_Cl_2_ solvate molecules (Figure 5). The CH_2_Cl_2_ molecules interact with the ladders through C—H···O hydrogen bonding as described above.

### Experimental

Eu(NO_3_)_3_ (0.051 g 0.15 mmol) was placed in the bottom of a test tube and covered with CH_2_Cl_2_ (5 ml). *4,4*'-bipyridine-*N,N*'-dioxide^.^H_2_O (0.0376 g, 0.182 mmol) was dissolved in methanol (8 ml), and this solution was layered over the CH_2_Cl_2_. The two solutions were allowed to slowly mix. Over a period of several weeks the Eu(NO_3_)_3 _dissolved, and colorless block-like crystals of the title compound formed.

### Refinement

All H atoms were positioned geometrically and refined using a riding model with C—H = 0.95 Å and with *U_iso_*(H) = 1.2 times *U*_eq_(C).

### Crystal data

**Table:**

[Eu_2_(NO_3_)_6_(C_10_H_8_N_2_O_2_)_3_]·2CH_2_Cl_2_	*Z* = 1
*M**_r_* = 1410.38	*F*(000) = 690
Triclinic, *P*1	*D*_x_ = 2.013 Mg m^−^^3^
Hall symbol: -P 1	Mo *K*α radiation, λ = 0.71073 Å
*a* = 7.9841 (5) Å	Cell parameters from 9986 reflections
*b* = 11.5723 (7) Å	θ = 2.4–31.4°
*c* = 13.0522 (8) Å	µ = 3.00 mm^−^^1^
α = 86.013 (1)°	*T* = 100 K
β = 80.255 (1)°	Block, colourless
γ = 78.392 (1)°	0.44 × 0.38 × 0.32 mm
*V* = 1163.45 (12) Å^3^	

### Data collection

**Table:**

Bruker SMART APEX CCD diffractometer	7017 independent reflections
Radiation source: fine-focus sealed tube	6748 reflections with *I* > 2σ(*I*)
graphite	*R*_int_ = 0.015
ω scans	θ_max_ = 31.5°, θ_min_ = 1.6°
Absorption correction: multi-scan (*SADABS*; Bruker, 2009)	*h* = −11→11
*T*_min_ = 0.278, *T*_max_ = 0.383	*k* = −16→16
13873 measured reflections	*l* = −18→18

### Refinement

**Table:**

Refinement on *F*^2^	Primary atom site location: structure-invariant direct methods
Least-squares matrix: full	Secondary atom site location: difference Fourier map
*R*[*F*^2^ > 2σ(*F*^2^)] = 0.020	Hydrogen site location: inferred from neighbouring sites
*wR*(*F*^2^) = 0.050	H-atom parameters constrained
*S* = 1.06	*w* = 1/[σ^2^(*F*_o_^2^) + (0.0274*P*)^2^ + 0.6833*P*] where *P* = (*F*_o_^2^ + 2*F*_c_^2^)/3
7017 reflections	(Δ/σ)_max_ = 0.004
334 parameters	Δρ_max_ = 1.30 e Å^−^^3^
0 restraints	Δρ_min_ = −0.90 e Å^−^^3^

### Special details

**Table:**

Geometry. All esds (except the esd in the dihedral angle between two l.s. planes) are estimated using the full covariance matrix. The cell esds are taken into account individually in the estimation of esds in distances, angles and torsion angles; correlations between esds in cell parameters are only used when they are defined by crystal symmetry. An approximate (isotropic) treatment of cell esds is used for estimating esds involving l.s. planes.
Refinement. Refinement of F^2^ against ALL reflections. The weighted R-factor wR and goodness of fit S are based on F^2^, conventional R-factors R are based on F, with F set to zero for negative F^2^. The threshold expression of F^2^ > 2sigma(F^2^) is used only for calculating R-factors(gt) etc. and is not relevant to the choice of reflections for refinement. R-factors based on F^2^ are statistically about twice as large as those based on F, and R- factors based on ALL data will be even larger.

### Fractional atomic coordinates and isotropic or equivalent isotropic displacement parameters (Å^2^)

**Table:**

	*x*	*y*	*z*	*U*_iso_*/*U*_eq_	
Eu1	0.777642 (10)	0.833489 (7)	0.717497 (6)	0.01106 (3)	
O1	1.02598 (16)	0.82745 (11)	0.59308 (10)	0.0154 (2)	
O2	0.95680 (16)	0.87385 (12)	0.83140 (9)	0.0161 (2)	
O3	0.62858 (17)	0.87321 (12)	0.57648 (9)	0.0171 (2)	
O4	0.80290 (19)	0.63688 (12)	0.64041 (12)	0.0232 (3)	
O5	0.95209 (19)	0.63704 (12)	0.76308 (11)	0.0220 (3)	
O6	0.9737 (2)	0.47449 (13)	0.68284 (15)	0.0332 (4)	
O7	0.48093 (17)	0.79129 (13)	0.77651 (10)	0.0201 (3)	
O8	0.64275 (17)	0.77354 (13)	0.89511 (10)	0.0202 (3)	
O9	0.37320 (17)	0.75544 (13)	0.93758 (11)	0.0227 (3)	
O10	0.80793 (18)	1.04165 (12)	0.66196 (10)	0.0194 (3)	
O11	0.59940 (17)	1.02059 (12)	0.78740 (11)	0.0195 (3)	
O12	0.6447 (3)	1.19617 (15)	0.73702 (16)	0.0456 (5)	
N1	1.15666 (18)	0.73751 (13)	0.56743 (11)	0.0133 (3)	
N2	0.92011 (18)	0.86855 (13)	0.93519 (11)	0.0132 (3)	
N3	0.69461 (19)	0.86783 (13)	0.47577 (11)	0.0137 (3)	
N4	0.9118 (2)	0.57887 (14)	0.69524 (14)	0.0197 (3)	
N5	0.49525 (19)	0.77188 (13)	0.87204 (12)	0.0150 (3)	
N6	0.6829 (2)	1.08969 (14)	0.72861 (13)	0.0205 (3)	
C1	1.1740 (3)	0.68713 (17)	0.47511 (14)	0.0203 (4)	
H1	1.0935	0.7159	0.4290	0.024*	
C2	1.3082 (3)	0.59415 (17)	0.44756 (14)	0.0206 (4)	
H2	1.3193	0.5593	0.3824	0.025*	
C3	1.4281 (2)	0.55034 (15)	0.51380 (13)	0.0136 (3)	
C4	1.4069 (2)	0.60727 (17)	0.60769 (14)	0.0183 (3)	
H4	1.4871	0.5816	0.6545	0.022*	
C5	1.2711 (2)	0.69995 (17)	0.63285 (14)	0.0188 (3)	
H5	1.2582	0.7376	0.6969	0.023*	
C6	0.9832 (2)	0.76980 (16)	0.98827 (14)	0.0158 (3)	
H6	1.0515	0.7039	0.9517	0.019*	
C7	0.9489 (2)	0.76417 (16)	1.09564 (14)	0.0162 (3)	
H7	0.9927	0.6941	1.1327	0.019*	
C8	0.8500 (2)	0.86102 (15)	1.14977 (13)	0.0130 (3)	
C9	0.7874 (2)	0.96184 (15)	1.09182 (13)	0.0147 (3)	
H9	0.7201	1.0294	1.1265	0.018*	
C10	0.8226 (2)	0.96384 (15)	0.98487 (13)	0.0151 (3)	
H10	0.7784	1.0323	0.9459	0.018*	
C11	0.7453 (2)	0.96291 (16)	0.42433 (13)	0.0166 (3)	
H11	0.7435	1.0314	0.4610	0.020*	
C12	0.7997 (2)	0.96088 (16)	0.31839 (13)	0.0161 (3)	
H12	0.8336	1.0286	0.2821	0.019*	
C13	0.8053 (2)	0.86034 (15)	0.26417 (13)	0.0126 (3)	
C14	0.7613 (3)	0.76130 (16)	0.32116 (14)	0.0184 (3)	
H14	0.7700	0.6898	0.2872	0.022*	
C15	0.7053 (3)	0.76727 (17)	0.42647 (14)	0.0198 (3)	
H15	0.6739	0.7000	0.4649	0.024*	
C16	0.5593 (3)	0.60128 (19)	1.10281 (18)	0.0274 (4)	
H16A	0.5804	0.6067	1.0258	0.033*	
H16B	0.5400	0.6821	1.1285	0.033*	
Cl1	0.74307 (7)	0.51437 (4)	1.14770 (4)	0.02594 (10)	
Cl2	0.37189 (7)	0.54009 (6)	1.14595 (5)	0.03328 (12)	

### Atomic displacement parameters (Å^2^)

**Table:**

	*U*^11^	*U*^22^	*U*^33^	*U*^12^	*U*^13^	*U*^23^
Eu1	0.01154 (4)	0.01311 (4)	0.00801 (4)	−0.00167 (3)	−0.00067 (3)	−0.00117 (3)
O1	0.0136 (5)	0.0141 (5)	0.0158 (6)	0.0005 (4)	0.0027 (4)	−0.0023 (4)
O2	0.0181 (6)	0.0241 (6)	0.0067 (5)	−0.0068 (5)	0.0001 (4)	−0.0016 (4)
O3	0.0172 (6)	0.0261 (7)	0.0070 (5)	−0.0033 (5)	0.0003 (4)	−0.0003 (5)
O4	0.0248 (7)	0.0175 (6)	0.0291 (7)	−0.0008 (5)	−0.0117 (6)	−0.0038 (5)
O5	0.0253 (7)	0.0188 (6)	0.0216 (7)	0.0000 (5)	−0.0075 (5)	−0.0028 (5)
O6	0.0305 (8)	0.0156 (6)	0.0535 (11)	0.0042 (6)	−0.0140 (8)	−0.0097 (7)
O7	0.0183 (6)	0.0307 (7)	0.0133 (6)	−0.0089 (5)	−0.0042 (5)	0.0008 (5)
O8	0.0147 (6)	0.0315 (7)	0.0159 (6)	−0.0088 (5)	−0.0042 (5)	0.0060 (5)
O9	0.0154 (6)	0.0277 (7)	0.0220 (7)	−0.0046 (5)	0.0030 (5)	0.0063 (5)
O10	0.0206 (6)	0.0181 (6)	0.0168 (6)	−0.0015 (5)	0.0019 (5)	−0.0005 (5)
O11	0.0192 (6)	0.0187 (6)	0.0179 (6)	−0.0011 (5)	0.0024 (5)	−0.0014 (5)
O12	0.0585 (12)	0.0154 (7)	0.0507 (12)	0.0006 (7)	0.0168 (9)	−0.0023 (7)
N1	0.0121 (6)	0.0134 (6)	0.0132 (6)	−0.0018 (5)	0.0015 (5)	−0.0024 (5)
N2	0.0126 (6)	0.0196 (7)	0.0086 (6)	−0.0060 (5)	−0.0007 (5)	−0.0018 (5)
N3	0.0141 (6)	0.0183 (7)	0.0085 (6)	−0.0026 (5)	−0.0019 (5)	0.0000 (5)
N4	0.0167 (7)	0.0158 (7)	0.0260 (8)	−0.0026 (6)	−0.0020 (6)	−0.0020 (6)
N5	0.0141 (6)	0.0145 (6)	0.0155 (7)	−0.0026 (5)	−0.0010 (5)	0.0013 (5)
N6	0.0234 (8)	0.0168 (7)	0.0187 (7)	0.0001 (6)	−0.0008 (6)	−0.0004 (6)
C1	0.0230 (9)	0.0223 (9)	0.0137 (8)	0.0040 (7)	−0.0053 (7)	−0.0051 (7)
C2	0.0232 (9)	0.0232 (9)	0.0143 (8)	0.0023 (7)	−0.0052 (7)	−0.0077 (7)
C3	0.0141 (7)	0.0146 (7)	0.0122 (7)	−0.0036 (6)	−0.0003 (6)	−0.0025 (6)
C4	0.0157 (8)	0.0239 (9)	0.0146 (8)	0.0005 (7)	−0.0035 (6)	−0.0062 (7)
C5	0.0161 (8)	0.0239 (9)	0.0161 (8)	−0.0005 (7)	−0.0026 (6)	−0.0081 (7)
C6	0.0159 (7)	0.0172 (7)	0.0137 (7)	−0.0018 (6)	−0.0016 (6)	−0.0024 (6)
C7	0.0172 (8)	0.0162 (7)	0.0139 (7)	−0.0006 (6)	−0.0024 (6)	−0.0010 (6)
C8	0.0129 (7)	0.0167 (7)	0.0095 (7)	−0.0035 (6)	−0.0012 (5)	−0.0002 (6)
C9	0.0161 (7)	0.0151 (7)	0.0113 (7)	−0.0002 (6)	−0.0008 (6)	−0.0014 (6)
C10	0.0171 (7)	0.0162 (7)	0.0112 (7)	−0.0025 (6)	−0.0014 (6)	0.0012 (6)
C11	0.0213 (8)	0.0155 (7)	0.0129 (7)	−0.0038 (6)	−0.0018 (6)	−0.0022 (6)
C12	0.0210 (8)	0.0161 (7)	0.0116 (7)	−0.0058 (6)	−0.0011 (6)	−0.0006 (6)
C13	0.0120 (7)	0.0147 (7)	0.0103 (7)	−0.0010 (6)	−0.0014 (5)	−0.0007 (6)
C14	0.0277 (9)	0.0154 (8)	0.0126 (8)	−0.0049 (7)	−0.0027 (6)	−0.0018 (6)
C15	0.0292 (9)	0.0181 (8)	0.0133 (8)	−0.0087 (7)	−0.0025 (7)	0.0012 (6)
C16	0.0267 (10)	0.0251 (10)	0.0280 (10)	−0.0022 (8)	−0.0047 (8)	0.0073 (8)
Cl1	0.0292 (2)	0.0207 (2)	0.0281 (2)	−0.00046 (18)	−0.01003 (19)	−0.00127 (18)
Cl2	0.0274 (2)	0.0405 (3)	0.0291 (3)	−0.0046 (2)	−0.0018 (2)	0.0059 (2)

### Geometric parameters (Å, °)

**Table:**

Eu1—O3	2.3279 (13)	C1—H1	0.9500
Eu1—O1	2.3332 (12)	C2—C3	1.395 (2)
Eu1—O2	2.3579 (12)	C2—H2	0.9500
Eu1—O11	2.4781 (13)	C3—C4	1.400 (2)
Eu1—O7	2.4979 (13)	C3—C3^i^	1.479 (3)
Eu1—O8	2.4994 (13)	C4—C5	1.376 (2)
Eu1—O5	2.5061 (14)	C4—H4	0.9500
Eu1—O4	2.5090 (14)	C5—H5	0.9500
Eu1—O10	2.5137 (14)	C6—C7	1.381 (2)
Eu1—N6	2.9160 (16)	C6—H6	0.9500
Eu1—N5	2.9271 (15)	C7—C8	1.394 (2)
Eu1—N4	2.9424 (16)	C7—H7	0.9500
O1—N1	1.3331 (18)	C8—C9	1.398 (2)
O2—N2	1.3365 (18)	C8—C13^ii^	1.475 (2)
O3—N3	1.3316 (18)	C9—C10	1.376 (2)
O4—N4	1.276 (2)	C9—H9	0.9500
O5—N4	1.268 (2)	C10—H10	0.9500
O6—N4	1.220 (2)	C11—C12	1.378 (2)
O7—N5	1.2717 (19)	C11—H11	0.9500
O8—N5	1.2680 (19)	C12—C13	1.393 (2)
O9—N5	1.220 (2)	C12—H12	0.9500
O10—N6	1.270 (2)	C13—C14	1.395 (2)
O11—N6	1.276 (2)	C13—C8^iii^	1.475 (2)
O12—N6	1.217 (2)	C14—C15	1.374 (2)
N1—C5	1.344 (2)	C14—H14	0.9500
N1—C1	1.349 (2)	C15—H15	0.9500
N2—C6	1.348 (2)	C16—Cl1	1.767 (2)
N2—C10	1.351 (2)	C16—Cl2	1.773 (2)
N3—C11	1.345 (2)	C16—H16A	0.9900
N3—C15	1.349 (2)	C16—H16B	0.9900
C1—C2	1.376 (3)		
			
O3—Eu1—O1	85.10 (4)	C5—N1—C1	120.97 (15)
O3—Eu1—O2	154.66 (5)	O2—N2—C6	119.71 (14)
O1—Eu1—O2	83.73 (4)	O2—N2—C10	118.95 (14)
O3—Eu1—O11	86.31 (5)	C6—N2—C10	121.33 (15)
O1—Eu1—O11	122.68 (4)	O3—N3—C11	119.85 (14)
O2—Eu1—O11	80.76 (5)	O3—N3—C15	119.01 (15)
O3—Eu1—O7	72.54 (4)	C11—N3—C15	121.12 (15)
O1—Eu1—O7	151.35 (4)	O6—N4—O5	122.25 (17)
O2—Eu1—O7	123.72 (4)	O6—N4—O4	122.21 (17)
O11—Eu1—O7	74.46 (5)	O5—N4—O4	115.54 (15)
O3—Eu1—O8	123.44 (4)	O6—N4—Eu1	177.07 (15)
O1—Eu1—O8	148.50 (4)	O5—N4—Eu1	57.72 (9)
O2—Eu1—O8	74.50 (4)	O4—N4—Eu1	57.89 (9)
O11—Eu1—O8	76.41 (5)	O9—N5—O8	122.22 (16)
O7—Eu1—O8	51.03 (4)	O9—N5—O7	121.86 (15)
O3—Eu1—O5	125.27 (5)	O8—N5—O7	115.90 (14)
O1—Eu1—O5	79.17 (5)	O9—N5—Eu1	174.99 (12)
O2—Eu1—O5	74.59 (5)	O8—N5—Eu1	58.05 (8)
O11—Eu1—O5	144.99 (5)	O7—N5—Eu1	58.00 (8)
O7—Eu1—O5	99.04 (5)	O12—N6—O10	122.03 (18)
O8—Eu1—O5	73.32 (5)	O12—N6—O11	121.27 (17)
O3—Eu1—O4	74.83 (5)	O10—N6—O11	116.70 (15)
O1—Eu1—O4	78.66 (5)	O12—N6—Eu1	177.57 (16)
O2—Eu1—O4	124.69 (5)	O10—N6—Eu1	59.16 (9)
O11—Eu1—O4	150.55 (5)	O11—N6—Eu1	57.57 (9)
O7—Eu1—O4	78.35 (5)	N1—C1—C2	120.10 (17)
O8—Eu1—O4	95.14 (5)	N1—C1—H1	120.0
O5—Eu1—O4	50.81 (5)	C2—C1—H1	120.0
O3—Eu1—O10	76.78 (5)	C1—C2—C3	121.05 (17)
O1—Eu1—O10	71.43 (4)	C1—C2—H2	119.5
O2—Eu1—O10	78.12 (5)	C3—C2—H2	119.5
O11—Eu1—O10	51.46 (4)	C2—C3—C4	116.71 (16)
O7—Eu1—O10	118.58 (5)	C2—C3—C3^i^	121.90 (19)
O8—Eu1—O10	124.05 (5)	C4—C3—C3^i^	121.39 (19)
O5—Eu1—O10	141.62 (5)	C5—C4—C3	120.72 (17)
O4—Eu1—O10	140.06 (5)	C5—C4—H4	119.6
O3—Eu1—N6	81.12 (5)	C3—C4—H4	119.6
O1—Eu1—N6	96.98 (5)	N1—C5—C4	120.42 (16)
O2—Eu1—N6	77.77 (5)	N1—C5—H5	119.8
O11—Eu1—N6	25.77 (4)	C4—C5—H5	119.8
O7—Eu1—N6	96.99 (5)	N2—C6—C7	120.21 (16)
O8—Eu1—N6	100.24 (5)	N2—C6—H6	119.9
O5—Eu1—N6	152.34 (5)	C7—C6—H6	119.9
O4—Eu1—N6	155.82 (5)	C6—C7—C8	120.18 (16)
O10—Eu1—N6	25.70 (4)	C6—C7—H7	119.9
O3—Eu1—N5	97.96 (4)	C8—C7—H7	119.9
O1—Eu1—N5	164.53 (4)	C7—C8—C9	117.82 (15)
O2—Eu1—N5	98.86 (4)	C7—C8—C13^ii^	123.07 (15)
O11—Eu1—N5	72.75 (4)	C9—C8—C13^ii^	119.09 (15)
O7—Eu1—N5	25.58 (4)	C10—C9—C8	120.41 (16)
O8—Eu1—N5	25.50 (4)	C10—C9—H9	119.8
O5—Eu1—N5	86.78 (5)	C8—C9—H9	119.8
O4—Eu1—N5	87.47 (5)	N2—C10—C9	120.05 (16)
O10—Eu1—N5	124.04 (4)	N2—C10—H10	120.0
N6—Eu1—N5	98.47 (4)	C9—C10—H10	120.0
O3—Eu1—N4	100.07 (5)	N3—C11—C12	119.90 (16)
O1—Eu1—N4	76.92 (4)	N3—C11—H11	120.0
O2—Eu1—N4	99.44 (5)	C12—C11—H11	120.0
O11—Eu1—N4	160.06 (5)	C11—C12—C13	120.50 (16)
O7—Eu1—N4	89.34 (5)	C11—C12—H12	119.7
O8—Eu1—N4	84.39 (5)	C13—C12—H12	119.7
O5—Eu1—N4	25.32 (5)	C12—C13—C14	117.84 (15)
O4—Eu1—N4	25.52 (5)	C12—C13—C8^iii^	120.62 (15)
O10—Eu1—N4	148.34 (5)	C14—C13—C8^iii^	121.50 (15)
N6—Eu1—N4	173.61 (5)	C15—C14—C13	119.88 (16)
N5—Eu1—N4	87.60 (4)	C15—C14—H14	120.1
N1—O1—Eu1	129.42 (10)	C13—C14—H14	120.1
N2—O2—Eu1	125.13 (10)	N3—C15—C14	120.59 (17)
N3—O3—Eu1	127.65 (10)	N3—C15—H15	119.7
N4—O4—Eu1	96.59 (10)	C14—C15—H15	119.7
N4—O5—Eu1	96.97 (10)	Cl1—C16—Cl2	111.26 (12)
N5—O7—Eu1	96.43 (10)	Cl1—C16—H16A	109.4
N5—O8—Eu1	96.46 (10)	Cl2—C16—H16A	109.4
N6—O10—Eu1	95.15 (10)	Cl1—C16—H16B	109.4
N6—O11—Eu1	96.66 (10)	Cl2—C16—H16B	109.4
O1—N1—C5	119.59 (14)	H16A—C16—H16B	108.0
O1—N1—C1	119.42 (15)		

Symmetry codes: (i) −*x*+3, −*y*+1, −*z*+1; (ii) *x*, *y*, *z*+1; (iii) *x*, *y*, *z*−1.

### Hydrogen-bond geometry (Å, °)

**Table:**

*D*—H···*A*	*D*—H	H···*A*	*D*···*A*	*D*—H···*A*
C5—H5···O7^iv^	0.95	2.41	3.081 (2)	128.
C9—H9···O9^v^	0.95	2.57	3.286 (2)	132.
C12—H12···O2^vi^	0.95	2.44	3.309 (2)	152.
C16—H16B···O12^v^	0.99	2.42	3.242 (3)	140.
C16—H16A···O8	0.99	2.55	3.307 (3)	133.
C16—H16A···O9	0.99	2.50	3.086 (3)	118.

Symmetry codes: (iv) *x*+1, *y*, *z*; (v) −*x*+1, −*y*+2, −*z*+2; (vi) −*x*+2, −*y*+2, −*z*+1.
